# Determinants of Herpetofaunal Diversity in a Threatened Wetland Ecosystem: A Case Study of the Ramaroshan Wetland Complex, Western Nepal

**DOI:** 10.3390/ani13010135

**Published:** 2022-12-29

**Authors:** Janaki Paudel, Laxman Khanal, Naresh Pandey, Laxmi Prasad Upadhyaya, Chandra Bahadur Sunar, Bina Thapa, Chet Raj Bhatta, Ramesh Raj Pant, Randall C. Kyes

**Affiliations:** 1 Central Department of Zoology, Institute of Science and Technology, Tribhuvan University, Kirtipur, Kathmandu 44618, Nepal; 2 Central Department of Environmental Science, Institute of Science and Technology, Tribhuvan University, Kirtipur, Kathmandu 44618, Nepal; 3 Aishwarya Multiple Campus, Tribhuvan University, Dhangadhi 10901, Nepal; 4 Departments of Psychology, Global Health, and Anthropology, Center for Global Field Study, and Washington National Primate Research Center, University of Washington, Seattle, WA 98195, USA

**Keywords:** herpetofauna, land use and land cover, amphibians, reptiles, wetland

## Abstract

**Simple Summary:**

Wetlands of mid-hills and lesser Himalaya in Nepal are highly threatened due to climate change and human encroachment. This study assessed the change in total surface coverage of water bodies in the Ramaroshan Wetland Complex of western Nepal over the last three decades and explored the diversity and environmental determinants of the resident herpetofauna by line transect sampling during September/October 2021. The wetland complex is threatened by a net loss of 16% in water coverage over the last three decades. Eleven species of herpetofauna (five amphibians and six reptiles) within five families of two orders were recorded in the study area. The area has low herpetofaunal diversity that indicates poor ecosystem health of the wetland complex. Therefore, we recommend immediate conservation and restoration initiatives to ensure the sustainability of the wetland.

**Abstract:**

Wetlands are among the highly threatened ecosystems due to anthropogenic activities. The Ramaroshan Wetland Complex (RWC) of Achham District, Nepal is one of the high-altitude wetlands facing human induced degradation and loss. Herpetofauna are key bio-indicators of environmental health and habitat quality and are useful to assess habitat conditions of such threatened ecosystems. This study quantified the land use and land cover (LULC) change in the RWC and documented the diversity and distribution pattern of herpetofauna. The LULC in the area (13.94 Km^2^) was analyzed for 1989, 2000, 2010 and 2021 by supervised classification of remote sensing images. Surveys were conducted along 25 transects, each of 200 m in length and environmental variables were recorded for every observation of herpetofauna. The LULC analysis revealed an overall loss of 16% of the total water body between 1989 (0.25 Km^2^) and 2021 (0.21 Km^2^). Eleven species of herpetofauna (five amphibians and six reptiles) within five families and two orders (i.e., Anura and Squamata), were recorded with low diversity (H’ = 1.88312) and evenness (E = 0.3642) indices. The herpetofauna had a hump-shaped distribution along the elevation gradient with the highest richness and abundance at 2300 m asl. Amphibian abundance decreased with increasing distance to nearest water sources, whereas reptile abundance increased. Amphibians were more abundant in agricultural field and marsh land, whereas reptile abundance was higher around human settlements. Results indicate that the wetland area in the RWC is declining at an alarming rate and, in turn, might account for the low diversity and abundance of the herpetofauna.

## 1. Introduction

Wetlands are among Earth’s most productive ecosystems. They play essential roles in regulating global climate, maintaining hydrologic cycles and blue carbon, protecting ecosystem diversity, disaster risk reduction and ensuring human wellbeing [1,2,3,4]. Despite their importance, wetlands are under threat due to their vulnerability and attractiveness to human development [5,6] and the majority have already undergone some form of degradation [7,8]. The primary pressures are not only habitat loss and degradation, but also reduced water inflow due to damming of rivers, deterioration of water quality due to excessive nutrient influx and organic matter resulting from factors, such as urbanization, industrial waste, agriculture, pollution and land reclamation [7,9,10]. Globally, the loss of natural wetlands averaged about 30% between 1970–2008, and the decline is accelerating annually [8,11].

Wetlands, with transitional characteristics of both terrestrial and aquatic habitats, harbor high biological diversity that is disproportionate to the area and are therefore known as biodiversity hotspots [12,13]. Among all taxa, herpetofauna are highly sensitive and depend heavily on the diverse wetland for breeding, foraging and refugia in winter [14]. Globally, wetlands harbor about 43.4% of the world’s herpetofaunal diversity [15]. However, several herpetofaunal species have already gone extinct or have suffered drastic reduction in their numbers due to degradation of wetland ecosystems [16,17,18,19]. Changes in availability and distribution of critical habitats, such as wetlands, result in the reduction in herpetofaunal diversity and abundance [20]. Biological indicators can be employed to obtain qualitative data to assess the ecological resources [21] and presence of herpetofauna (reptiles and amphibians) in an ecosystem can be used to assess the environmental conditions [22]. Herpetofauna are excellent candidates for indicator species of wetland health due to the dependence of amphibians on water and the habitat specialization of many reptiles [21]. Therefore, assessing the distribution of wetlands and monitoring its dynamic changes is of utmost importance.

Geospatial analysis of wetlands using remote sensing and GIS is one of the recent approaches for the assessment of wetland spatial and temporal distribution and its quality [23,24]. Remote sensing images of fine spatial and hyperspectral resolution have been widely used in mapping wetlands [25,26]. Landsat collections (such as Landsat-5 TM, Landsat-7 ETM+ and Landsat-8 OLI) using geo-referenced images acquired by optical imagery are an effective and efficient means of studying wetland habitats [27]. Through these methods, it is possible to obtain an accurate landscape-scale assessment of wetland status and study its trends without having to conduct time-consuming and expensive fieldwork [28,29].

Nepal, a small landlocked Himalayan country that occupies only 0.003% of the world’s area, has 10 sites designated as wetlands of international importance (Ramsar Sites) with a total surface area of 605.61 km^2^. These sites are home for about 27% of the nationally threatened birds [30], 85% of endemic vertebrates [31] and 230 indigenous fish species [32]. Geographically, the wetlands of Nepal range from high-altitude to lowland. High-altitude wetlands, such as Gosainkunda, Gokyo, Rara and Phoksundo, provide provisioning services, such as freshwater, cultural services, such as tourism, spiritual and religious experiences. The mid-hills wetlands, such as- Mai Pokhari and Lake Clusters of Pokhara Valley, provide provisioning services, such as food and fuel. The lowland wetlands, such as Koshi Tappu, Jagadishpur, Beeshazari and Ghodaghodi, provide both cultural and provisioning services [33,34]. Despite their considerable significance for human and ecosystem support, the wetlands of Nepal are under tremendous anthropogenic pressure [35].

The Ramaroshan Wetland Complex (RWC), a landscape of 12 lakes and 18 meadows, is situated in western Nepal [36]. It is one of the potential Ramsar sites with natural and permanent water bodies [37] and biological diversity including globally threatened birds [38]. The area is facing considerable threats such as gulley erosion, over grazing and land conversion. Lakes, such as Lisse Daali and Geraha Lake, have been converted into grassland and cropland [36]. These natural and artificial factors have reduced the wetland habitat. Given these increasing pressures, a baseline study exploring the current status of the water bodies and biodiversity in the RWC is deemed essential. Management action plans that consider small vertebrates, such as herpetofauna, as biological indicators are crucial for the sustainability of the RWC. However, to date, a systematic study to document herpetofaunal diversity has yet to be conducted. Therefore, this research was conducted to (i) investigate the land use pattern and land cover change in the RWC, (ii) assess the herpetofaunal community structure in the RWC and (iii) examine the effects of environmental variables on the herpetofaunal community.

## 2. Materials and Methods

### 2.1. Study Area

**Figure 1:**
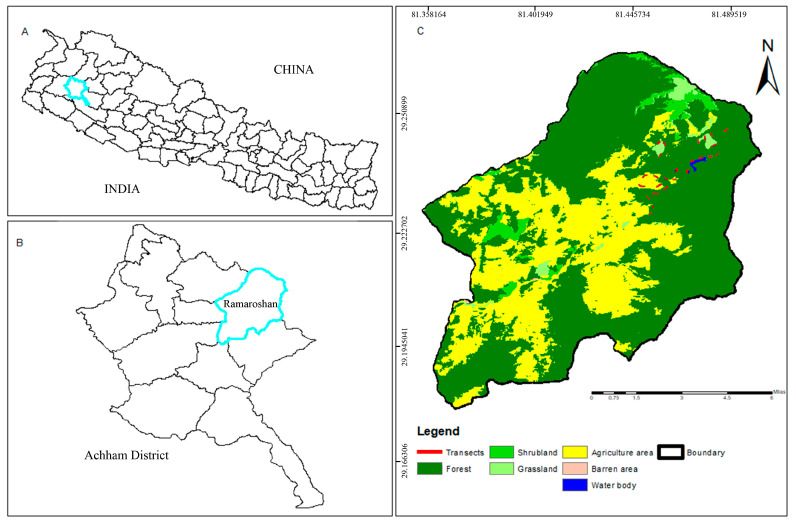
Map of the study area. (**A**) Map of Nepal showing Achham District; (**B**) Map of Achham District showing Ramaroshan Rural Municipality; and (**C**) Map of Ramaroshan Rural Municipality showing transects around the Ramaroshan Wetland Complex.

This study was conducted in the Ramaroshan Wetland Complex (RWC, Figure 1), a mid-hill rural wetland extending from 1401 m to 3792 m asl in Achham District of Sudurpashchim Province, Nepal. The RWC consists of 12 lakes and 18 meadows interconnecting with each other forming a complex [37]. The only outlet of the lake water is Kailash River [39]. The RWC lies 42 km from Mangalsen, the district headquarters of Achham District. Vegetation in the area is characterized by mid-hills flora, such as Rhododendron (*Rhododendron* spp.), Chestnut (*Castanopsis indica*), Needle-wood (*Schima walichii*), Alder (*Alnus nepalensis*), Pine (*Pinus roxburghii*), Himalayan yew (*Taxus wallichiana*), Moso bamboo (*Phyllostachys pubescens*), Lantana (*Lantana camara*), Mugwort (*Artemisia vulgaris*) and yellow Himalayan raspberry (*Rubus ellipticus*). Major fauna of the area includes Himalayan monal (*Lophophorous impejanus*), Kalij pheasant (*Lophura leucomelanos*), Mallard (*Anas platyrhynchos*), Black-headed jay (*Garrulus lanceolatus*) (a bird that has been recorded only in the far-western region of Nepal), Himalayan black bear (*Ursus thibetanus*), Musk deer (*Moschus chrysogaster*), Leopard (*Panthera pardus*), etc.

### 2.2. Herpetofaunal Survey

A reconnaissance survey was conducted during the third week of May 2021 in order to identify the wetlands and herpetofaunal survey areas. A line transect survey was conducted for the collection of data in and around the major wetlands of Ramaroshan area from 27 September to 07 October 2021. A total of 25 transects, each of 200 m lengths, were established along the walking trails in the area. The elevation of the survey ranged between 1400 m asl and 3800 m asl. Transect sampling was conducted daily along various transects from 4 PM to 10 PM. Every individual encountered within a distance of two meters on either side of a transect was captured, identified and counted (see below). Additionally, opportunistic captures and records were conducted throughout the day. Transects at each elevation band were surveyed over lakes, streams, forests, settlement areas and croplands whenever possible.

While walking along a transect, every encountered individual was photographed on the spot, collected in a ventilated jar, then identified using the field guide book “Herpetofauna of Nepal: A Conservation Companion” [40]. After identification, specimens were marked with a permanent marker to avoid repetition and released back. Unidentified individuals were euthanized with ethanol and tagged on the hind leg. All the tagged specimens were kept in absolute ethanol in a closed bottle and transferred to the laboratory of the Central Department of Zoology, Tribhuvan University for subsequent identification by experts with the aid of samples and photographs. Environmental variables, such as slope, aspect, distance to water, distance to human settlement, elevation and habitat type, were noted for each observation of the herpetofauna (Table 1).

### 2.3. Remote Sensing Data Acquisition and Land-Use Classification

Landsat images from 1989, 2000, 2010 and 2021 were used to detect LULC change. The Landsat 4–5 TM (Thematic mapper) for 1989, Landsat 7-ETM (Enhanced Thematic Mapper) for 2000, Landsat 5-TM for 2010 and Landsat 8-OLI (Operational Land Images) for 2021 with same spatial resolution (30 m) and with images taken during the first week of March were downloaded from United States Geological Survey (USGS) geoportal (https://glovis.usgs.gov/app, accessed on 15 July 2022). The entire Landsat images consisted of <10% of cloud cover. A total area of 13.94 km^2^ of the Ramaroshan Rural Municipality within a polygon of latitude 29.250899°, 29.251255°, 29.223386°, 29.222702° and longitude 81.445734°, 81.489519°, 81.489888° and 81.445793° was selected for the classification. Data sets were digitized by the ArcGIS tool, then land cover was categorized into five land-use types: i.e., vegetation, grassland, water bodies, barren land and agricultural land (Table 2). The supervised classification was performed following the methods employed by [41]. The signature classes or training samples were prepared from Google Earth map.

Accuracy assessment is an important part of any classification project. It compares the classified image to another data source that is considered to be accurate or ground truth data. Ground truth points were collected during the field work. Accuracy assessment was performed on the resulting classified imagery using error matrix and Kappa index to test the precision and accuracy of imagery and comparing them with actual points from the field. Ground truthing points (*n* = 30) were used as a reference for the accuracy assessment of the classified images of 2021. For Landsat images of 1989, 2000 and 2010, 90 stratified random points were generated and compared with the reference from Google Earth. The user’s accuracy, producer accuracy and overall accuracy were obtained from the error matrix. Kappa coefficient is the statistical evaluation of the classified map’s accuracy and is used to determine how precisely the agreements between model prediction and reality match [42]. The Kappa coefficient ranges from 0 to 1. A coefficient close to zero indicates no agreements, values between 0–0.2 denote mild agreements, 0.21–0.40 indicate fair agreements, 0.41–0.60 indicate moderate agreements, 0.61–0.80 indicate satisfactory or good agreements and 0.81–1 indicate almost perfect accord [43]. Accuracy of the classified images were calculated using following formulas:

$$\mathrm{User}’s\;\mathrm{Accuracy}=\frac{\mathrm{Number}\;\mathrm{of}\;\mathrm{correctly}\;\mathrm{classified}\;\mathrm{pixels}\;}{\mathrm{Total}\;\mathrm{number}\;\mathrm{of}\;\mathrm{classified}\;\mathrm{pixels}\;\mathrm{in}\;\mathrm{that}\;\mathrm{category}\;\left(\mathrm{Row}\;\mathrm{Total}\right)}\times 100$$

$$\mathrm{Producer}\;\mathrm{Accuracy}=\frac{\mathrm{Number}\;\mathrm{of}\;\mathrm{correctly}\;\mathrm{classified}\;\mathrm{pixels}}{\mathrm{Total}\;\mathrm{number}\;\mathrm{of}\;\mathrm{reference}\;\mathrm{pixels}\;\mathrm{in}\;\mathrm{that}\;\mathrm{category}\left(\mathrm{Column}\;\mathrm{Total}\right)}\times 100$$

$$\mathrm{Overall}\;\mathrm{Accuracy}=\frac{\;\mathrm{Total}\;\mathrm{number}\;\mathrm{of}\;\mathrm{correctly}\;\mathrm{classified}\;\mathrm{pixels}\left(\mathrm{Diagonal}\right)}{\mathrm{Total}\;\mathrm{Number}\;\mathrm{of}\;\mathrm{Reference}\;\mathrm{Pixels}}\times 100$$

$$\mathrm{Kappa}\;\mathrm{Coefficient}=\frac{\left(\mathrm{TS}\times \mathrm{TCS}\right)-\Sigma \left(\mathrm{Column}\;\mathrm{Total}\times \mathrm{Row}\;\mathrm{Total}\right)}{{\mathrm{TS}}^{2}-\Sigma \left(\mathrm{Column}\;\mathrm{Total}\times \mathrm{Row}\;\mathrm{Total}\right)}\times 100$$

where, TS = Total Sample and TCS = Total Corrected Sample

### 2.4. Herpetofaunal Data Analysis

The Shannon–Wiener diversity index (H’) and evenness index (E) of the herpetofauna were calculated using PAST version 3.5 [44]. The generalized linear model (GLM) was performed to test the effects of environmental variables on the abundance of herpetofauna using R-studio [45]. A redundancy analysis (RDA) was employed in CANOCO version 4.5 [46] to examine association between distribution of herpetofauna and environmental variables. Final results were presented in the form of a biplot with a Monte Carlo permutation test by using 499 permutations to identify which variables had a significant effect on the distribution of amphibians and reptiles in study area.

## 3. Results and Discussion

### 3.1. Land Use and Land Cover Change in the RWC

The land use and land cover classification of the RWC from 1989 to 2021 showed variation in the land-use classes over the time. The overall accuracies of the classified images for 1989, 2000, 2010 and 2021 were 70%, 80%, 83.33% and 86.66%, respectively (Supplementary Table S1). The user’s accuracy ranged from 50% to 83.33% in 1989, 60% to 85.71% in 2000, 66.66% to 89.97% in 2010 and 71.42% to 91% in 2021. The kappa coefficients for the years 1989, 2000, 2010 and 2021 were 0.62, 0.74, 0.79 and 0.83 respectively.

**Figure 2:**
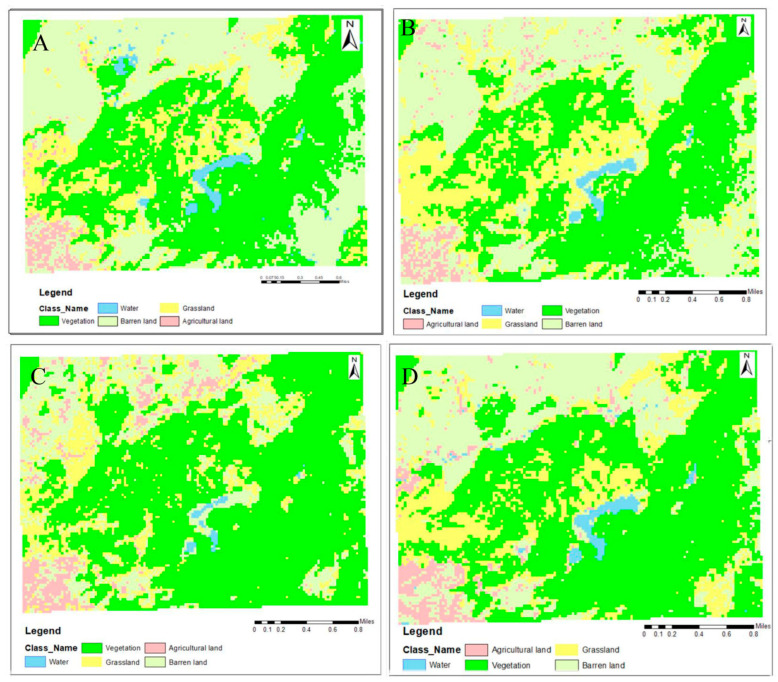
Patterns of land use and land cover from 1989–2021. (**A**) LULC of the RWC for 1989; (**B**) LULC of the RWC for 2000; (**C**) LULC of the RWC for 2010; and (**D**) LULC of the RWC for 2020.

The results of the analysis of the 1989 image (Figure 2, Table 3) shows that vegetation, barren land and grassland dominated the landscape with 36.7%, 31.6% and 26.6%, respectively. Compared to 1989, analysis of the 2000 image showed that vegetation and barren land remained constant, whereas the water bodies decreased by 0.1% and the agricultural land increased by 0.5% of the total study area. Similarly, the 2010 image, when compared to that of 2000, showed that vegetation and agricultural land increased, grassland remained almost constant, and barren land and water bodies decreased. Finally, from 2010 to 2021, vegetation and agricultural land increased; barren land and grassland continued to decrease and water bodies showed a modest increase. However, there was a net decrease in water bodies from 1989 (0.25 km^2^) to 2021 (0.21 km^2^) that accounted for a 16% loss.

Using the initial land cover data (from 1989) as a baseline, the patterns of LULC change analysis demonstrated the direction of land cover changes. Classified images of the RWC showed a 16% decrease in water bodies; perhaps due to overexploitation of the area by the local people. The lake named Dallena in the RWC was converted to farmland by the local people during Maoist insurgency period from 1995 to 2005/2006. The largest decrease by proportion to 1989 is the grassland coverage likely due to overgrazing, which has a significant environmental impact on the soil’s preservation, diversity and water holding capacity [47,48,49].The increase in agricultural land may be due to the return of the local people who had been displaced by severe flooding of the Kailash River in the 2020. The degradation of a water body necessitates immediate management action, especially given the ongoing climate change-related droughts that are occurring worldwide [50]. On account of people moving from rural to urban areas to seek a higher quality of life with greater economic opportunities, the cropland left behind gradually turns into forest [51] albeit lower quality, secondary forest. The observed decrease in barren area in the RWC might be due to the replacement by forest. The grassland also showed a progressive reduction, which may be because locals use the grassland in the mountains as pasture land [52]. Interestingly, the coverage of vegetation in the RWC area showed an increase in 2021, which can be attributed to the commendable support from the Ramaroshan Tourism Board and the local government.

### 3.2. Herpetofaunal Community Structure of the RWC

A total of 179 individuals of herpetofauna belonging to 11 species (5 amphibians and 5 reptiles) within five families of two orders were recorded in the study area (Table 4, Supplementary Figure S1). All five species of amphibians belonged to order Anura and the reptiles belonged to the order Squamata. Dicroglossidae was the most dominant family consisting of three species followed by Bufonidae consisting of two species. The relative abundance of *Duttaphrynus melanostictus* was the highest (0.284) and that of *Elaphe hodgsonii* and *Nanorana rostandi* was the least (0.005).

**Figure 3:**
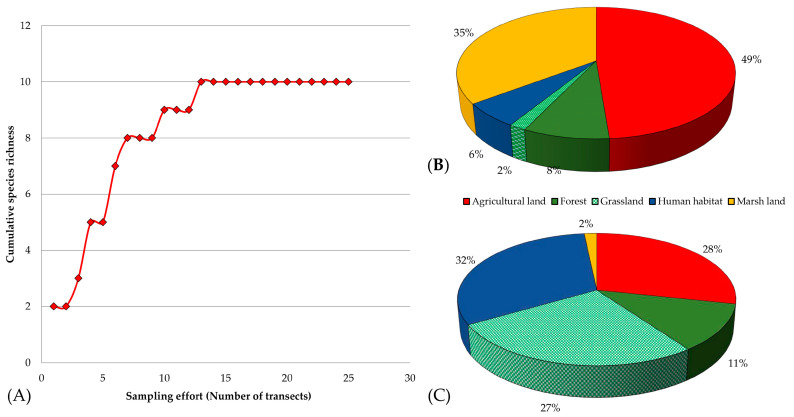
Herpetofaunal community structure in different habitats of the RWC; (**A**) Species accumulation curve of herpetofauna; (**B**) The abundance of amphibians in different habitats; (**C**) The abundance of reptiles in different habitats.

The overall Shannon–Weiner diversity index (H’) of herpetofauna was 1.88312 and the evenness index (E) was 0.3642. The diversity index (H’) and evenness index for amphibians were 1.2892 and 0.6211, respectively and for the reptiles, 1.2825 and 0.7213, respectively. The species accumulation curve reached an asymptote (Figure 3A), indicating that there were no prospects of finding new species even with increased sampling efforts [53]. The presence of 179 individuals of 11 species (5 amphibians and 6 reptiles) in this field study implies that the herpetofaunal diversity is likely impacted by the time of study period (post monsoon). Due to the minimal rainfall during the post-monsoon season, species discovery may have been limited. Similar findings of reduced herpetofaunal diversity in pre-monsoon season have been noted by others [54]. Among the 11 species of herpetofauna identified, *Duttaphrynus melanostictus* was the most abundant species in the area. However, according to local people, the area also has tree frogs which were not observed during this study. The family Dicroglossidae was largest for amphibians and the family Scincidae was largest for reptiles. *Calotes versicolar* was recorded at an altitude of 2540 m asl in this study. Previous reports have identified this species in a variety of terrestrial environments below 2000 m asl [40].

Habitat types determine herpetofaunal species diversity and community structure [55]. The highest abundance of amphibians was observed in agricultural land (49%) followed by the marsh land (Figure 3B). This may be because amphibians tend to congregate around water sources where there is a greater abundance prey. Notably, however, multiple studies have demonstrated that agricultural contamination (e.g., pesticides and insecticides) and habitat loss owing to expansion of road systems are key drivers in global amphibian decrease [56,57]. Limited use of pesticides by the local subsistence farmers might have provided suitable habitats for the amphibians in the RWC. Higher diversity and lesser conservation risks were reported for amphibians in the agricultural fields of the Democratic People’s Republic of Korea [58]. The grassland had lowest abundance of amphibians, probably due to limited kinds of food options and less diverse habitat. Reptiles were most abundant near the human settlement (Figure 3C). Agamids had higher relative abundance and such habitat generalist reptiles have been reported to thrive well in and around human settlements [59,60]. The higher abundance of reptiles in human habitat, grassland and agricultural areas of the RWC might be associated with availability of higher structural diversity, opportunity for higher insolation and escape than in dense forest or marsh land areas.

### 3.3. Effects of Environmental Variables on Abundance of Herpetofauna

**Figure 4:**
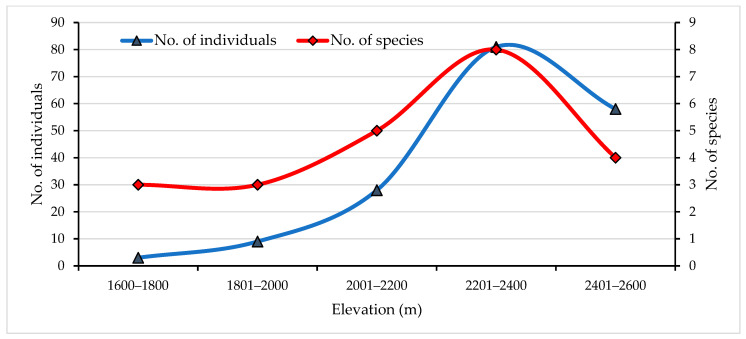
Herpetofauna species richness and abundance along elevational gradients in the RWC.

This study revealed a hump-shaped distribution of the herpetofauna along the elevation gradient of the RWC (Figure 4). Herpetofaunal species richness and abundance gradually increased with increasing elevation that peaked at 2201–2400 m asl and dropped off beyond that. The hump shaped relationship between species richness and elevation in this study might be because the RWC’s major wetlands are situated at 2100–2500 m asl, providing both wet and dry conditions for herpetofaunal assemblage which is fundamental for their various life stages. Additionally, many amphibians have relocated their ranges towards higher elevations due to the growing effects of global warming and climate change [61,62]. Similar altitudinal richness patterns have been documented on the diversity of plants, frogs, lizards, snakes, birds and small mammals along elevation gradients of Himalaya and adjoining mountain rages [63,64,65,66,67,68,69]. However, a monotonic decline in amphibian richness and abundance also has been recorded from the eastern Nepal Himalayas [70] which could be due to a majority of recorded amphibians inhabiting a narrow elevational range.

Amphibians showed a statistically significant negative association with distance to water source (Table 4), suggesting that amphibian abundance tends to decline with increasing distance from water bodies. Amphibians are associated with moist environments [71,72]. As a proxy for water availability, soil moisture is a crucial predictor of amphibian occurrence [73]. The reptile abundance was positively linked with distance to water and negatively associated with elevation (Table 5). Greater species richness of lizards at higher elevations in southern latitudes have been reported because high elevations in southern latitudes experience exceptional variation in seasonal temperatures and soil moisture, which seem to benefit the physiological needs of lizards [74]. However, the cool, dry higher elevations of the Himalayan region support a smaller diversity of reptiles [54,72]. Distance to human settlements had a significant effect on herpetofauna in the RWC where both amphibian and reptile species were associated with distance from the nearest human settlement. Of all the 11 species of herpetofauna, *L. tuberculata* was found most frequently in the human habitats, such as on the roofs of houses and in holes in houses. This may be due to a greater availability of prey in and around the human habitat.

**Figure 5:**
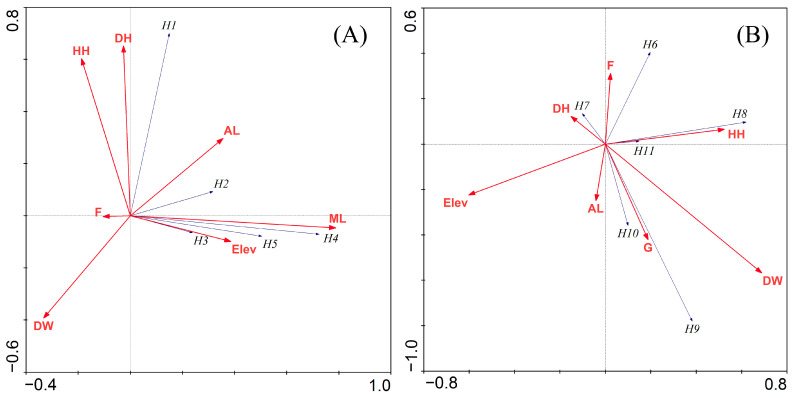
RDA ordination diagram (biplot) showing the association of environmental variables with the herpetofauna in the RWC; (**A**) Amphibians; and, (**B**) Reptiles. Note: F = Forest, ML = Marsh Land, AL = Agricultural Land, G = Grassland, HH = Human Habitat, DW = Nearest distance to water resource, Elev = Elevation, DH = Nearest distance to human settlement.

The relation of environmental variables on the species richness of amphibians was tested for selected habitat types including agricultural land, forest, human habitat and marsh land. A Monte Carlo permutation test of significance of all the reduced model axes revealed a significance preference of the amphibian species (Trace = 0.384, F = 3.118 and *p* = 0.0120) to different habitat types and environmental variables with marsh land showing the greatest association. (Figure 5A). Similarly, reptilian species also showed a significant preference (Trace = 0.448, F = 1.967 and *p* = 0.05) with the greatest associations with forest, human habitat and grassland (Figure 5B). The RDA ordination diagram showed that *Duttaphrynus himalayanus* was more associated with human habitat. *D. melanostictus* was associated with agricultural land. *Polunini paa*, *Nanorana minica* and *Nanorana rostandi* were more associated with marsh land. *Elaphe hodgsonii* was more associated with the forest area. *Laudakia tuberculata* and *Asymblepharus ladacensis* were more associated with human habitat area. *Calotes versicolar* and *Asymblepharus himalayanus* were more associated with grassland. Among the habitat variables, amphibian and reptilian abundances had higher associations with elevation and distance to human habitat, respectively.

The research had some limitations with respect to the study period. Due to inaccessibility of the area during the monsoon season, field surveys were conducted only during the post-monsoon period. Therefore, the lower diversity of herpetofauna might be associated with the limited survey period. Nevertheless, the data on the herpetofauna and their habitat are crucial and will serve as a baseline standard for any further research in the region. Due to its varied habitat and anthropogenic activities altering the land use pattern and threatening the ecosystem, the biodiversity in the RWC demands immediate conservation initiatives. An extensive herpetofaunal survey covering all seasons is important for a more complete assessment and will play a critical role in developing comprehensive baseline measures and implementing conservation action in the RWC.

## 4. Conclusions

This study revealed that the LULC change around the RWC is the result of human encroachment and the growing demand for settlement, development and agriculture expansion. The ecosystem is threatened with a 16% loss in wetland area from 1989 to 2021, with corresponding low diversity of herpetofauna—an important indicator species of ecosystem health. Amphibians depicted strong association with marsh land and their abundance decreased with an increase in distance to water sources. Reptiles showed robust association with forest, human settlement and grassland and had higher abundance further from wetlands. Owing to the anthropogenic activities altering the land use pattern and threatening the ecosystem, we recommend immediate steps be taken to implement biodiversity conservation initiatives. The findings of this study provide important baseline data needed to design effective conservation and management strategies of the threatened wetlands.

## Figures and Tables

**Table 1:** Environmental variables recorded in the field and their description.

Parameter	Variables	Description	Codes Used
Habitat	Habitat type	Forest, marsh land, agricultural land, grassland, human habitat	F, ML, AL, G, HH
	Nearest distance to water resource	Euclidean distance measured from sampling location to the closest waterhole	DW
Topography	Elevation	Elevation (meter) above sea level	Elev
Disturbance	Nearest distance to human settlement	Euclidean distance measured from sampling points to nearest human settlements	DH

Note: F = Forest, ML = Marsh Land, AL = Agricultural Land, G = Grassland, HH = Human Habitat, DW = Nearest distance to water resource, Elev = Elevation, DH = Nearest distance to human settlement.

**Table 2:** Major land use and land cover types classified in the RWC.

S.N.	Land Cover Types	Description
1	Water body	Lakes, marsh land, river
2	Barren land	Dry places, flood plains without vegetation, landslide and no vegetation areas
3	Grassland	Meadows and irregular bushes
4	Vegetation	Forested area, mixed forest type
5	Agricultural land	Crop (e.g., paddy, maize, millet, etc.) cultivated land

**Table 3:** Spatial extent of land cover classes in RWC.

Land Use and Land Cover Classes	Year								Net Change
	1989		2000		2010		2021		
	Area (Km^2^)	Area (%)	Area (Km^2^)	Area (%)	Area (Km^2^)	Area (%)	Area (Km^2^)	Area (%)	
Agricultural land	0.46	3.3	0.53	3.8	0.76	5.5	0.78	5.6	Increased
Barren land	4.40	31.6	4.39	31.5	4.08	29.2	4.07	29.2	Decreased
Grassland	3.71	26.6	3.68	26.4	3.61	25.9	3.49	25.0	Decreased
Vegetation	5.12	36.7	5.10	36.6	5.30	38.0	5.39	38.7	Increased
Water body	0.25	1.8	0.24	1.7	0.19	1.4	0.21	1.5	Decreased
Total	13.94	100	13.94	100	13.94	100	13.94	100	

**Table 4:** List of herpetofaunal species recorded in the RWC with their relative abundance and species code used for redundancy analysis.

Taxa	Species	Family	Order	Relative Abundance	Code
Amphibians	*Duttaphrynus himalayanus*	Bufonidae	Anura	0.201	H1
	*Duttaphrynus melanostictus*	Bufonidae	Anura	0.284	H2
	*Nanorana minica*	Dicroglossidae	Anura	0.067	H3
	*Nanorana polunini*	Dicroglossidae	Anura	0.106	H4
	*Nanorana rostandi*	Dicroglossidae	Anura	0.005	H5
Reptiles	*Calotes versicolar*	Agamidae	Squamata	0.072	H9
	*Laudakia tuberculata*	Agamidae	Squamata	0.178	H8
	*Amphiesma platyceps*	Colubridae	Squamata	0.011	H7
	*Elaphe hodgsonii*	Colubridae	Squamata	0.005	H6
	*Asymblepharus himalayanus*	Scincidae	Squamata	0.016	H10
	*Asymblepharus ladacensis*	Scincidae	Squamata	0.050	H11

**Table 5:** Summary of GLM showing the effects of environmental variables on the abundance of herpetofauna in the RWC.

Herpetofauna	Variables	Estimate	SE	Z	*p*
Amphibians	Intercept	1.1223	1.9323	0.581	0.561
	Elevation	0.0005	0.0008	0.673	0.501
	Nearest distance to water	−0.0097	0.0023	−4.231	2.32 × 10^−5^ *
	Nearest distance to settlement	0.0001	0.0001	1.407	0.160
Reptiles	Intercept	4.0049	1.4716	2.752	0.0059 *
	Elevation	−0.0015	0.0006	−2.430	0.0150 *
	Nearest distance to water	0.0005	0.0001	3.466	0.0005 *
	Nearest distance to settlement	−0.0005	0.0004	−1.278	0.2013

Note: * = statistically significant.

## Data Availability

The data used in the study will be made available upon the request to the corresponding author.

## Supplementary Materials

The following supporting information can be downloaded at: https://www.mdpi.com/article/10.3390/ani13010135/s1, Table S1. Accuracy assessment of the classified images. Figure S1. Photographs of herpetofauna recorded from the Ramaroshan Wetland Complex, Achham District, Nepal.
