# Supplementary material for: Determinants of Herpetofaunal Diversity in a Threatened Wetland Ecosystem: A Case Study of the Ramaroshan Wetland Complex, Western Nepal
Source: Animals (Basel). 2022 Dec 29;13(1):135. doi: 10.3390/ani13010135 (PMC9817512; doi:10.3390/ani13010135)
Supplement: Supplementary file 1 [file animals-13-00135-s001.zip › animals-2018231-supplementary.pdf]

Determinants of Herpetofaunal Diversity in a Threatened Wetland Ecosystem: A Case Study of the Ramaroshan Wetland Complex, Western Nepal

Janaki Paudel [1, †], Laxman Khanal [1 * †], Naresh Pandey [1], Laxmi Prasad Upadayaya [1], Chandra Bahadur Sunar [1], Bina Thapa [2], Chet Raj Bhatta [3], Ramesh Raj Pant [2], Randall C. Kyes [4]

Supplementary Materials

Supplementary Table S1. Accuracy assessment of the classified images.

Land cover	1989		2000		2010		2021	
	UA	PA	UA	PA	UA	PA	UA	PA
**Agricultural land**	80	50	60	75	89.97	62.5	87.33	62.5
**Barren land**	71.42	71.42	85.71	75	85.71	100	71.42	100
**Grassland**	66.66	66.66	83.33	71.42	66.66	80	83.33	83.33
**Vegetation**	50	96.23	83.33	100	83.33	91.34	91	94
**Water body**	83.33	83.33	83.33	83.33	83.33	83.33	83.33	92.56
**Overall accuracy**	70		80		83.33		86.66	
**Kappa coefficient**	0.62		0.74		0.79		0.83	

Notes: UA- User Accuracy, PA- Producer's accuracy

Supplementary Figure S1. Photographs of herpetofauna recorded from the Ramaroshan Wetland Complex, Achham District, Nepal

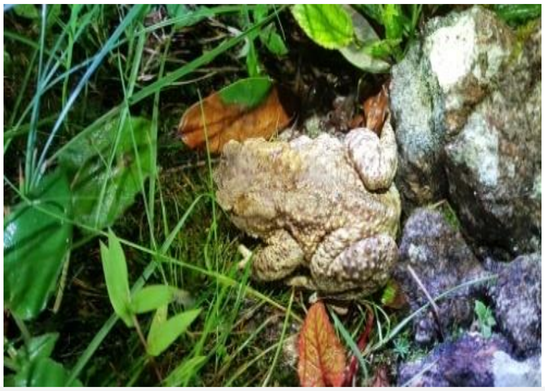

Photo 1: ***Duttaphrynus himalayanus***

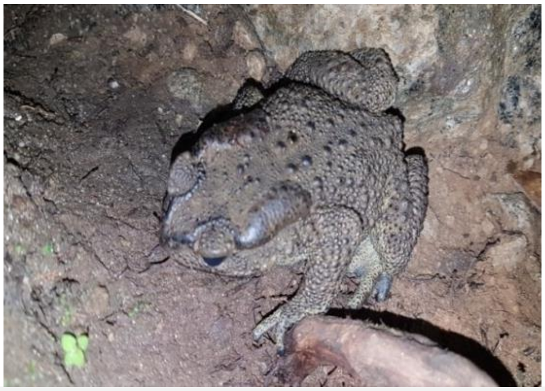

Photo 2: ***D. melanostictus***

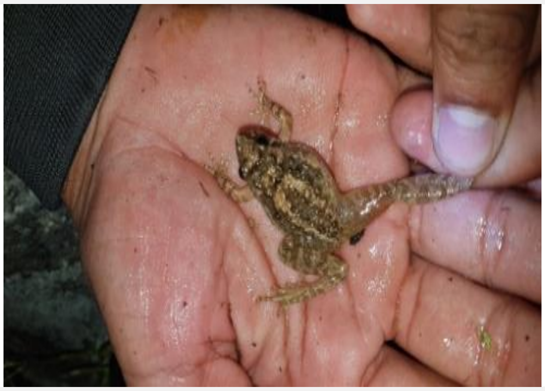

Photo 3: ***Nanorana minica***

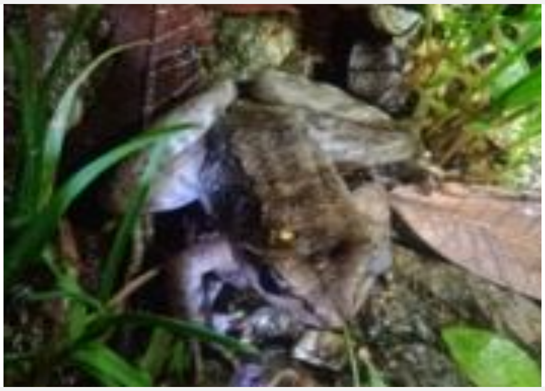

Photo 4: ***Polunin paa***

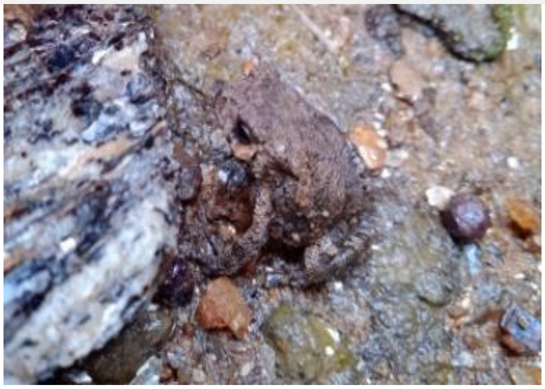

Photo 5: ***Nanorana rostandi***

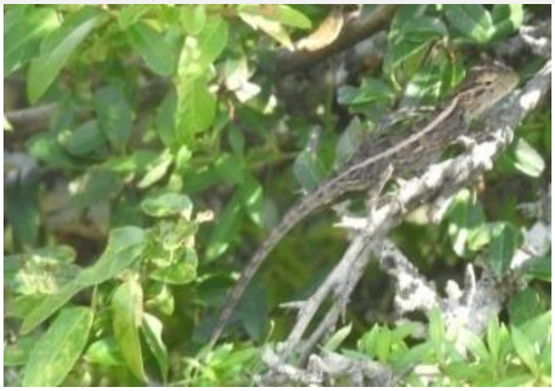

Photo 6: ***Calotes versicolor***

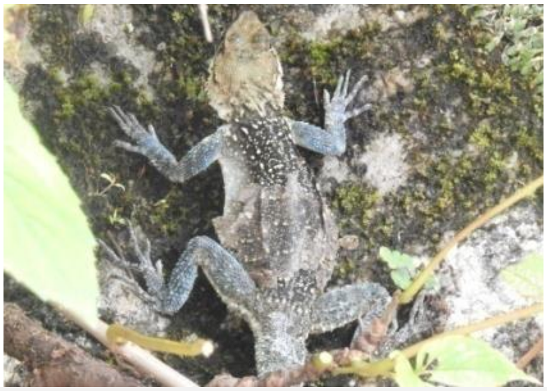

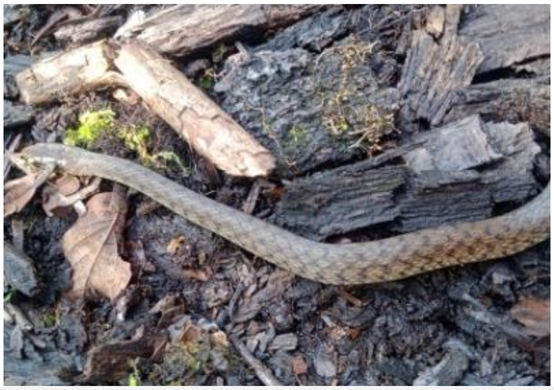

Photo 7: ***Laudakia tuberculata*** **Photo 8:** ***Elaphe hodgsonii***

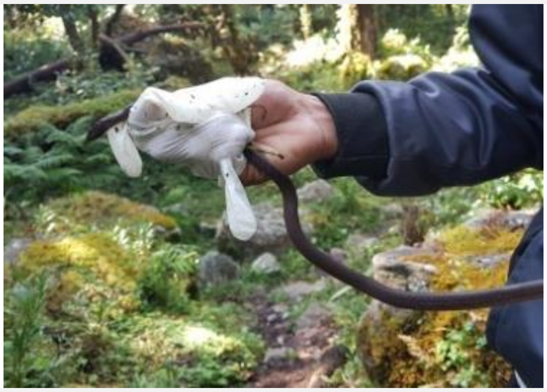

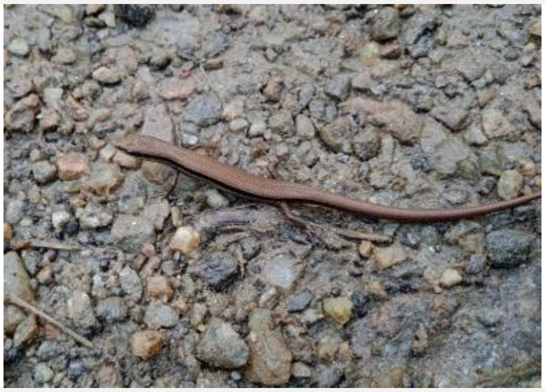

Photo 9: ***Amphiesma platyceps*** **Photo 10:** ***Asymblepharus ladacensis***

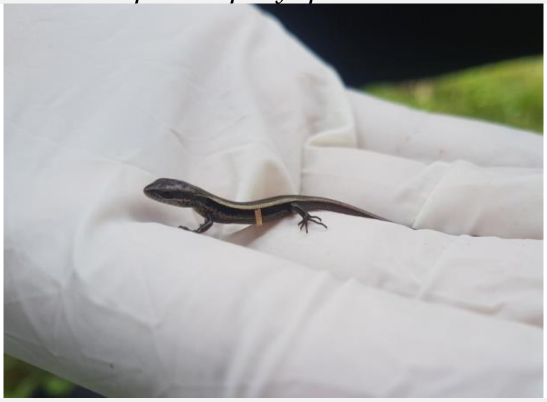

Photo 11: ***A. himalayanus***
